# Early Detection of Pancreatic Cancer: Current Advances and Future Opportunities

**DOI:** 10.3390/biomedicines13071733

**Published:** 2025-07-15

**Authors:** Zijin Lin, Esther A. Adeniran, Yanna Cai, Touseef Ahmad Qureshi, Debiao Li, Jun Gong, Jianing Li, Stephen J. Pandol, Yi Jiang

**Affiliations:** 1 College of Osteopathic Medicine, Michigan State University, East Lansing, MI 48067, USA; linzijin@msu.edu; 2 Samuel Oschin Comprehensive Cancer Institute, Cedars-Sinai Medical Center, Los Angeles, CA 90048, USA; esther.adeniran@cshs.org; 3 Peking Union Medical College Hospital, Beijing 100730, China; s2023001077@pumc.edu.cn (Y.C.); jli516@jh.edu (J.L.); 4 Biomedical Imaging Research Institute, Cedars-Sinai Medical Center, Los Angeles, CA 90048, USA; touseefahmad.qureshi@cshs.org (T.A.Q.); debiao.li@cshs.org (D.L.); 5 Division of Medical Oncology, Cedars-Sinai Medical Center, Los Angeles, CA 90048, USA; jun.gong@cshs.org; 6 Division of Gastroenterology, Johns Hopkins Medical Institutions, Baltimore, MD 21287, USA; 7 Karsh Division of Gastroenterology, Departments of Medicine, Cedars-Sinai Medical Center, Los Angeles, CA 90048, USA; stephen.pandol@cshs.org

**Keywords:** pancreatic ductal adenocarcinoma, early detection, risk stratification, biomarkers, radiomics, artificial intelligence

## Abstract

Pancreatic ductal adenocarcinoma (PDAC) remains among the most lethal malignancies, with a five-year survival rate below 12%, largely attributable to its asymptomatic onset, late-stage diagnosis, and limited curative treatment options. Although PDAC accounts for approximately 3% of all cancers, it is projected to become the second leading cause of cancer-related mortality in the United States by 2030. A major contributor to its dismal prognosis is the lack of validated early detection strategies for asymptomatic individuals. In this review, we present a comprehensive synthesis of current advances in the early detection of PDAC, with a focus on the identification of high-risk populations, novel biomarker platforms, advanced imaging modalities, and artificial intelligence (AI)-driven tools. We highlight high-risk groups—such as those with new-onset diabetes after age 50, pancreatic steatosis, chronic pancreatitis, cystic precursor lesions, and hereditary cancer syndromes—as priority populations for targeted surveillance. Novel biomarker panels, including circulating tumor DNA (ctDNA), miRNAs, and exosomes, have demonstrated improved diagnostic accuracy in early-stage disease. Recent developments in imaging, such as multiparametric MRI, contrast-enhanced endoscopic ultrasound, and molecular imaging, offer improved sensitivity in detecting small or precursor lesions. AI-enhanced radiomics and machine learning models applied to prediagnostic CT scans and electronic health records are emerging as valuable tools for risk prediction prior to clinical presentation. We further refine the Define–Enrich–Find (DEF) framework to propose a clinically actionable strategy that integrates these innovations. Collectively, these advances pave the way for personalized, multimodal surveillance strategies with the potential to improve outcomes in this historically challenging malignancy.

## 1. Introduction

Pancreatic ductal adenocarcinoma (PDAC) is a relatively rare but devastating malignancy that accounts for over 90% of pancreatic cancer cases. While pancreatic cancer includes several rare subtypes, this review focuses on PDAC due to its predominant histology and disproportionate clinical burden. Despite representing only about 3% of all cancers diagnosed, PDAC is responsible for approximately 8% of cancer-related mortality in the United States [1]. In 2025 alone, the American Cancer Society estimates over 67,000 new cases of PDAC and nearly 51,000 deaths, reflecting a mortality burden disproportionate to its incidence [1]. It is expected to rise to the second leading cause of cancer death by 2030, surpassing even breast and colorectal cancer [2]. In contrast to the declining mortality seen in many other malignancies, death rates for pancreatic cancer have steadily risen over the past century. In the early 1900s, mortality was approximately 5 per 100,000 in both men and women; today, it has climbed to 13 per 100,000 in men and 10 per 100,000 in women [1]. This rise partly reflects improvements in reporting, but also an increase in incidence, potentially linked to population aging, obesity, and type 2 diabetes (T2DM). This high mortality is primarily driven by insidious progression, resistance to traditional cancer therapies, and the lack of a validated strategy for early detection in the general population [3].

The prognosis of PDAC is closely associated with the stage at diagnosis. Over 50% of patients are diagnosed with advanced or metastatic disease, where curative surgical resection is no longer an option [4]. In such cases, patients primarily receive palliative care, including systemic chemotherapy or multimodal pain control [4]. Fewer than 10–15% of patients present with localized, clearly resectable tumors where surgery is recommended based on guidelines [1]. Consequently, the overall 5-year survival rate for PDAC remains dismal at 3–15% [1,4]. Median survival for metastatic disease is typically measured in months, whereas early-stage detection can dramatically shift this trajectory.

The concept of early detection for pancreatic cancer has evolved from historically identifying symptomatic disease or significant tumor burden—by which point the optimal window for curative intervention may have already passed—to targeting an asymptomatic, screen-detectable phase. For pancreatic cancer specifically, lesions such as pancreatic intraepithelial neoplasia (PanINs), mucinous cystic neoplasms (MCNs), and intraductal papillary mucinous neoplasms (IPMNs) may be present for years before progressing to PDAC [5]. While PanINs remain a challenge to detect clinically, IPMNs are increasingly identified incidentally through cross-sectional imaging and are now considered actionable precursor lesions in high-risk individuals.

However, widespread screening in average-risk populations is currently not recommended due to the low incidence of PDAC and high likelihood of false positives, which could lead to unnecessary surgical intervention and associated psychological and physical burden [6]. Instead, focus has shifted to surveillance in high-risk groups—individuals with a strong family history of PDAC, known germline mutations (e.g., *BRCA1/2*, *PALB2*, *CDKN2A*), pancreatic cystic lesions, or new-onset diabetes mellitus after the age of 50 [7].

This review builds upon prior foundational work in early detection of PDAC by providing a state-of-the-art synthesis of developments from the past five years. Compared to earlier reviews [7,8], our review incorporates recent advances in emerging tools such as AI-enabled imaging, pancreatic fat as a risk phenotype, multi-omics biomarkers, and electronic health record–based prediction models. We also refine the Define–Enrich–Find framework to offer a more clinically actionable approach. Taken together, the aim of this review is to synthesize current knowledge and highlight recent advances in the early detection of PDAC, identify research gaps, and propose a multidisciplinary approach to improve early diagnosis and survival outcomes.

To form this review, we conducted a comprehensive literature search using databases including PubMed, Scopus, and Google Scholar. Our search strategy focused on identifying peer-reviewed publications relevant to the early detection and diagnosis of pancreatic ductal adenocarcinoma (PDAC). We included original research articles, systematic reviews, meta-analyses, and established clinical guidelines. Studies were selected based on their relevance to one or more of the following inclusion criteria: (1) novel diagnostic strategies or biomarker development for PDAC, (2) clinical evaluation of imaging- or molecular-based detection tools, (3) risk stratification methods in high-risk or genetically predisposed populations, and (4) insights into the pathophysiology underlying early pancreatic tumorigenesis. Preference was given to studies published within the past five years to reflect the most current advancements in the field.

## 2. Challenges and Emerging Risk-Based Approaches in Early Detection

Early detection of PDAC remains a longstanding challenge due to several factors. First, the pancreas’s deep anatomical location renders tumors challenging to detect during routine physical examinations. Furthermore, early-stage pancreatic cancer often presents with nonspecific or absent symptoms, leading to diagnosis at advanced stages when curative treatment is no longer feasible [9].

Second, practical diagnostic tools are lacking. The low incidence of PDAC in the general population, combined with the limited sensitivity of computed tomography (CT) and magnetic resonance imaging (MRI) in identifying small or early lesions, contributes to a low positive predictive value for any screening modality. The high cost of these imaging methods, and in the case of CT, radiation exposure, further limit their feasibility for widespread screening. The United States Preventive Services Task Force (USPSTF) has assigned a Grade D recommendation against screening for PDAC in asymptomatic, average-risk individuals, citing evidence that not only is routine screening unhelpful, but it may lead to harm due to unnecessary interventions and anxiety [6]. Additionally, while carbohydrate antigen 19-9 (CA19-9) remains a commonly utilized biomarker, its sensitivity and specificity are insufficient for reliable early detection [10].

Third, the absence of clearly defined high-risk populations further complicates the implementation of targeted screening programs. Most biomarker assays—including protein panels, autoantibodies, circulating tumor DNA (ctDNA), methylated DNA, microRNAs, and exosomes—have demonstrated limited diagnostic accuracy, especially in the asymptomatic surveillance setting [11]. Nevertheless, they have shown potential in diagnosing patients in earlier stages of pancreatic cancer.

Collectively, these challenges underscore the urgent need for cost-effective and non-invasive strategies to enhance early detection and ultimately improve patient outcomes in pancreatic cancer [8]. To address these limitations, the Define–Enrich–Find (DEF) framework has been proposed to implement early detection in selected populations at a higher risk of PDAC. This model begins by defining individuals at elevated risk based on clinical, familial, and genetic criteria. The second step, enrich, involves applying clinical risk models or stratification tools, such as biomarkers, imaging modalities, or machine learning algorithms, to further narrow the at-risk group. The final step, find, refers to the application of screening methods, ranging from high-resolution imaging to liquid biopsy and artificial intelligence (AI)-enhanced radiomics to detect early, asymptomatic disease [12]. It is important to note that in practice, the boundary between the “Enrich” and “Find” stages is not always rigid. Many tools, particularly biomarkers and imaging technologies, can serve dual functions. For instance, a biomarker or imaging tool may both flag high-risk individuals and directly detect early disease. Acknowledging such overlap underscores the framework’s flexibility and clinical relevance.

To operationalize the DEF framework in primary care, a tiered, risk-adaptive approach may be used: the “Define” step can be implemented using electronic health record (EHR)-integrated risk flags (e.g., age > 50 with new-onset diabetes, family history, or known pancreatic cysts). The “Enrich” step may involve non-invasive biomarker panels or clinical prediction tools to further refine the risk pool. Based on individual risk, biomarker testing can be performed at intervals of 6 to 12 months, with shorter intervals (e.g., every 3 to 6 months) considered for those with concerning trends. Recent guidelines from the American Society for Gastrointestinal Endoscopy (ASGE) emphasize the importance of personalized, risk-based strategies in screening high-risk individuals. As these risk-adapted strategies mature, the DEF framework can serve as a practical roadmap for integrating early detection into real-world primary care settings. As advancements in biomarker discovery, AI, and health record data integration continue, a multidisciplinary detection strategy may finally enable earlier diagnosis and improved survival in this otherwise devastating disease [13].

## 3. Define High-Risk Individuals

### 3.1. New-Onset Diabetes

The relationship between PDAC and metabolic dysregulation is complex and bidirectional. One of the most well-established risk markers for PDAC is new-onset T2DM, particularly in adults over the age of 50. A large-scale, population-based study conducted in China over an eight-year period found that individuals with new-onset T2DM had a more than five-fold increased risk of developing pancreatic cancer, with causality supported by Mendelian randomization analysis in an East Asian population [14]. This association was consistent with prior findings from U.S. cohorts, such as the Nurses’ Health Study (NHS) and Health Professionals Follow-Up Study, where a more than two-fold increase in risk was observed in both recent-onset diabetes and longstanding diabetes [15]. To quantify relative risk, the study used the standardized incidence ratio (SIR), which compares the observed number of cancer cases in a study population to the number expected based on incidence rates in a reference population. Using this metric, the highest relative risk was observed among individuals with young-onset T2DM (age 20–54), who had an SIR of 5.73. Furthermore, both longer diabetes duration (>5 years) and poor glycemic control (fasting blood glucose ≥ 10.0 mmol/L) were independently associated with elevated risk, with the highest SIR of 16.73 observed in those with early-onset diabetes and severe hyperglycemia [14].

To better differentiate pancreatic cancer-associated diabetes (PC-NOD) from routine T2DM, researchers developed the END-PAC (Enriching New-Onset Diabetes for Pancreatic Cancer) score to predict pancreatic cancer risk by incorporating age, weight loss, and change in blood glucose. The model demonstrated that 75% of PC-NOD patients had an END-PAC score ≥ 3, whereas 50% of individuals with T2DM scored ≤0. In a population-based validation cohort, an END-PAC score of ≥3 significantly increases the incidence of PDAC from 0.8% to 3.6%, with a sensitivity of 78% and specificity of 82%, and identified 75% of subjects in the discovery cohort >6 months before a diagnosis of pancreatic cancer [16]. While this threshold effectively enriches for high-risk individuals, false positives remain a key consideration. In prospective evaluation, approximately 60% patients with Type 2 New-Onset Diabetes and END-PAC ≥ 3 had alternate explanations such as another malignancy, steroid-induced hyperglycemia, or severe illness. However, exclusion of such confounding conditions reduced false positives to 10% and substantially improved predictive performance [16]. Accordingly, we propose using END-PAC ≥ 3 as an initial enrichment tool, followed by a secondary triage using additional clinical or biomarker-based criteria (such as elevated CA19-9, pancreatic steatosis on imaging, or unexplained weight loss) prior to initiating invasive testing. This tiered approach aligns with the DEF framework and enhances specificity while preserving early detection sensitivity.

Other risk models are also emerging. A sequential clinical model was recently developed to predict PDAC in patients newly diagnosed with impaired fasting glucose (IFG), using data from over 138,000 individuals in the UK THIN database [17]. This model, which incorporated age, body mass index (BMI), Proton Pump Inhibitors (PPI) use, total cholesterol, low-density lipoprotein (LDL), alanine transaminase (ALT), and alkaline phosphatase, achieved acceptable discrimination when using a >0.5% risk threshold for recommending PDAC screening, yielding a sensitivity of 17% and specificity of 98%. Additionally, machine learning approaches are now being explored to enhance risk stratification. A recent study applying machine learning to large-scale datasets demonstrated improved predictive performance over traditional models. The top 1% and 10% of predicted risk groups captured around 13% and 44% of all subsequent pancreatic cancer cases, respectively [18].

### 3.2. Adipose Tissue and Pancreatic Steatosis

Pancreatic steatosis, or intra-pancreatic fat deposition (IPFD), refers to the accumulation of fat within the pancreas. The term “fatty pancreas” is also commonly used in the literature as a synonym for pancreatic steatosis. In this discussion, we use these terms interchangeably. Pancreatic steatosis and IPFD have gained increasing attention as a potential precursor or risk enhancer for PDAC. Once considered an incidental radiologic finding, IPFD is now recognized as a metabolic and inflammatory condition that may contribute to pancreatic carcinogenesis [19]. Recent research has begun to clarify its epidemiologic significance, pathophysiologic underpinnings, and potential value in early cancer risk stratification.

Over the past decade, epidemiology studies, including case–control and retrospective studies across diverse geographic regions, have consistently demonstrated a strong association between pancreatic steatosis and PDAC. Desai et al. used non-contrast CT to evaluate pancreatic steatosis, quantified via the P.S100 index (attenuation difference between pancreatic and spleen), and found it significantly more pronounced in PDAC cases than controls [odds ratio (OR) of 3.75, *p* = 0.00017] [20]. Similarly, Hoogenboom et al. analyzed CT scans of 149 patients and reported pancreatic steatosis in 71.9% of PDAC cases, compared to 45.3% of controls (adjusted OR 2.7, *p* = 0.037), and the degree of steatosis was more pronounced on CTs performed within six months of PDAC diagnosis [21]. Furthermore, Endoscopic ultrasound (EUS)-based studies showed that hyperechoic pancreatic parenchyma—indicative of fatty infiltration on imaging—was associated with significantly increased odds of PDAC, with ORs of 18.03 (*p* = 0.001) and 2.35 (*p* = 0.04), respectively [22,23]. More recently, a prospective cohort and Mendelian randomization (MR) study by Yamazaki et al. using UK Biobank data from 29,463 participants provided compelling evidence supporting a causal relationship, reporting that individuals with IPFD > 10% had a 3.35-fold increased risk of PDAC. By leveraging genome-wide association data and adjusting for BMI, the MR analysis demonstrated that IPFD was associated with an OR of 2.46 [95% confidence interval (CI): 1.38–4.40] for developing PDAC [24]. These findings suggest that the association between pancreatic fat and PDAC risk is likely independent of overall body fat distribution. In addition to its association with PDAC, pancreatic steatosis has been linked to the presence of precursor lesions such as PanINs and IPMNs. Imaging and histologic studies show a positive correlation between the extent of pancreatic fat content and the number of PanIN lesions and fibrosis [25,26]. Despite these emerging insights, the clinical interpretation of pancreatic steatosis remains challenging, in part due to the absence of standardized diagnostic criteria. For example, CT studies have used pancreatic attenuation values (Hounsfield units or pancreas-to-spleen ratios), while EUS-based studies have relied on subjective echogenicity comparisons with kidney or spleen [19,27].

Pancreatic steatosis can develop via two primary mechanisms: fatty infiltration and fatty replacement. Fatty infiltration involves ectopic accumulation of adipocytes within the pancreatic parenchyma, commonly driven by metabolic syndrome, sedentary lifestyles, and visceral obesity. It is more frequently associated with insulin resistance [28]. Fatty replacement, by contrast, is an irreversible process and occurs when adipocytes replace acinar cells following injury. This is seen in the context of alcohol abuse, chronic pancreatitis, hepatitis B virus infection, malnutrition, and exposure to medications such as corticosteroids and chemotherapeutics [29]. Despite strong epidemiologic evidence demonstrating an association between pancreatic steatosis and PDAC, the mechanistic pathways connecting fat accumulation to carcinogenesis remain incompletely understood [30]. One prevailing hypothesis suggests that steatosis may potentiate the progression of precursor lesions—such as PanINs and IPMNs—by creating a pro-inflammatory microenvironment that promotes oncogenic KRAS signaling [28]. Adipokine imbalance and immune dysregulation, including upregulation of IL-1, IL-6, and tumor necrosis factor-a, along with suppression of regulatory T cells, may act in concert with predisposing genetic mutations to promote neoplastic transformation [31]. Emerging pathways have further expanded our understanding of this association. Overactivation of the renin-angiotensin system (RAS) in adipose tissue and pancreatic cells promotes endoplasmic reticulum stress, fibrosis, and inflammation and is linked to tumor proliferation and metastasis [32]. Pancreatic stellate cells, when activated by inflammatory cytokines and growth factors, contribute to desmoplasia and tumor progression. Additionally, impaired mitophagy and oxidative stress—induced by fatty acid accumulation and dysregulated iron metabolism—may lead to DNA damage, acinar-ductal metaplasia, and tumorigenesis [33,34].

Despite accumulating evidence, pancreatic steatosis has not yet been incorporated into formal PDAC surveillance protocols. Therapeutically, IPFD represents a potentially reversible condition. Although studies examining the relationship between lifestyle interventions, IPFD reduction, and subsequent PDAC risk remain limited, bariatric surgery has emerged as a promising intervention. It has shown promise not only in reducing pancreatic steatosis but also in lowering PDAC risk. Prospective imaging studies further support this observation, showing substantial reductions in pancreatic fat content following surgery [35]. For example, one study found a 43.8% decrease in pancreatic steatosis and a 51.2% reduction in hepatic steatosis postoperatively [36]. Additionally, a large retrospective cohort study reported that individuals who underwent bariatric surgery had a significantly lower risk of developing pancreatic cancer [37].

### 3.3. Inflammation and “-Itis”

Recurrent pancreatic inflammation in chronic pancreatitis (CP) is a well-established contributor to pancreatic carcinogenesis [38]. The synergy between inflammation and oncogene activation drives malignant transformation [39]. The risk is further compounded by environmental factors, particularly tobacco smoking, and genetic predispositions such as germline mutations in *PRSS1* and *SPINK1* [40].

While the overall incidence of PDAC arising from CP remains relatively low, the cumulative risk in alcohol-related CP has been estimated at approximately 4% after 15–20 years of disease duration [39]. This risk is significantly higher in hereditary pancreatitis, with cumulative incidence reaching 19% and 12% by age 60 among patients with *PRSS1* and *SPINK1* mutations, respectively [39]. Moreover, idiopathic acute pancreatitis, particularly in older patients, has been increasingly recognized as a potential presenting manifestation of PDAC. In such cases, tumor-induced inflammation and ductal obstruction may be the underlying cause of pancreatitis, and surveillance imaging is warranted following an episode of unexplained pancreatitis [41].

Early detection of cancer in the context of CP remains extremely challenging. First, the overlapping clinical symptoms of PDAC and CP—such as back pain, jaundice, weight loss, and deteriorating glycemic control—complicate timely diagnosis. Secondly, imaging interpretations are complicated by structural remodeling and fibrosis inherent to CP, which can obscure neoplastic lesions [41]. Lastly, certain forms of CP, including paraduodenal pancreatitis, may mimic malignancy on imaging and even biopsy, leading to potential misdiagnosis [42].

To address these diagnostic challenges, short-interval follow-ups with imaging are recommended in high-risk CP patients, especially when new symptoms arise despite alcohol abstinence or post-cholecystectomy. EUS-guided fine-needle biopsy (FNB), combined with molecular testing for mutations in genes such as *KRAS*, *TP53*, *CDKN2A*, and *SMAD4*/*DPC4*, can enhance the differentiation between inflammatory and neoplastic processes [43,44].

### 3.4. Cystic Precursor Lesions

Cystic neoplasms of the pancreas represent one of the few well-characterized and clinically recognized progression pathways to PDAC. Among these, IPMNs and MCNs are of particular significance, as they are often incidentally detected on cross-sectional imaging and possess established malignant potential. Early detection and management of these cystic lesions offer one of the most promising avenues for intercepting PDAC at a preinvasive stage, when curative intervention is still feasible.

The prevalence rate of pancreatic cystic lesions (PCLs) was 16%, most of which were under 10 mm. Age-specific prevalence of PCLs increased from 9% at 50 to 59 years, to 18% at 60 to 69 years, 26% at 70 to 79 years, and 38% at 80 years and above [45]. The clinical challenge lies in distinguishing high-risk cysts from benign lesions. While main duct IPMNs and branch-duct IPMNs (BD-IPMNs) with high-risk features (main duct involvement, mural nodules, or size > 3 cm) are associated with increased malignancy risk, most BD-IPMNs do not progress to cancer. Recent retrospective data show that the overall risk of malignant transformation in BD-IPMNs remains approximately 8% even after 10 years of follow-up [46]. In fact, up to 25% of surgically resected branch-duct IPMNs show no evidence of high-grade dysplasia or invasive disease. In a large cohort of 363 patients with BD-IPMNs followed for over 5 years, malignancy occurred in around 5% of patients, and invasive cancer in 4.4%. Notably, five patients developed malignant transformation after 10 years of surveillance, emphasizing the need for ongoing monitoring in select cases [46]. Recent evidence-based guidelines recommend more advanced diagnostic evaluation by multidisciplinary teams for IPMN measuring greater than 30 mm [47,48].

Mucinous cystic neoplasms (MCNs) account for a smaller proportion (approximately 3%) of resected pancreatic lesions, with a strong predilection for middle-aged women. A recent cohort study from China reported a 2.8% prevalence of MCNs among resected pancreatic lesions, with nearly half diagnosed incidentally and a median age of 47.2 years [49]. Key predictors of malignant transformation include age ≥ 50 years, tumor size ≥ 4 cm, pancreatic duct dilation, and the presence of mural nodules or solid components [49]. Recent evidence and guidelines support a more adaptive conservative approach, especially in patients with small, asymptomatic lesions that do not exhibit high-risk features such as tumor size ≥ 3 cm, rapidly increasing in size, and mural nodules [47,48].

In addition to IPMNs and MCNs, several other types of pancreatic cysts are clinically relevant, each with distinct morphologic, histologic, and risk profiles. These include solid pseudopapillary neoplasms (SPNs), cystic pancreatic neuroendocrine neoplasms (PENs), serous cystadenomas, and pseudocysts. A summary of the key features and estimated malignancy risks for each cyst type is provided in Table 1 to aid in clinical differentiation and decision-making.

**Table 1:** Characteristics of Common Pancreatic Cysts and associated PDAC risks. Adapted from Gardner et al. [50] and Elta, G. H. [48], modified to include estimated PDAC risk. MD-IPMN: Main Duct Intraductal Papillary Mucinous Neoplasm, BD-IPMN: Branch Duct Intraductal Papillary Mucinous Neoplasm, MCN: Mucinous Cystic Neoplasm, PEN: Pancreatic Endocrine Neoplasm, SPN: Solid Pseudopapillary Neoplasm, CA19-9: Carbohydrate Antigen 19-9.

Cyst Type	Morphology [48]	Histology [50]	Patient Population [48]	Malignant Potential [48]	Estimated PDAC Risk (%) [50,51,52]
**MD-IPMN**	Segmental or widespread dilation of the main pancreatic duct	Mucin-secreting goblet cells within dysplastic columnar epithelium; subtypes include intestinal, pancreatobiliary, and oncocytic	Older adults, often with ductal dilation or elevated CA19-9	Yes (moderate risk)	6–45%
**BD-IPMN**	Localized to the side branches of the pancreatic duct without involving the main duct	Mucin-producing goblet cells lining dysplastic columnar epithelium, typically of gastric type	Common in the elderly, an incidental finding on imaging	Yes (very minimal risk)	3–15%
**MCN**	Single thick-walled cyst, typically located in the pancreatic body or tail	Lined by epithelial cells surrounded by dense stroma similar to ovarian tissue	Middle-aged women, especially perimenopausal	Yes (moderate risk)	10–20%
**Cystic PEN**	Mixture of cystic and solid areas, occasionally showing calcification along the outer edges	Derived from islet cells, expressing neuroendocrine markers	Adults with suspected neuroendocrine tumors	Yes (minimal risk)	Not applicable
**SPN**	Unilocular lesion with solid, cystic, and papillary components, often with areas of hemorrhage or necrosis	Displays degeneration with mixed solid and cystic areas, including papillary projections and hemorrhage	Young women (<40 years old)	Yes (moderate risk)	10–20%
**Serous cystadenoma**	Microcystic or honeycomb-like appearance; oligocystic variants are less common	Lined by cuboidal epithelium without mucin production; lacks significant atypia	Predominantly affects women in their 60s	No	0
**Pseudocyst**	Simple fluid collection that may contain debris, without a true epithelial lining	No true epithelial lining; composed of inflammatory cells and fibrous tissue	Associated with a history of acute or chronic pancreatitis	No	0

### 3.5. Familial and Inherited Risk

Up to 10% of PDAC cases occur in individuals with an inherited predisposition, broadly categorized into hereditary cancer syndromes and familial pancreatic cancer (FPC). Hereditary cancer syndromes include Hereditary Breast and Ovarian Cancer (HBOC; *BRCA1/2*, *ATM*, *PALB2*), Lynch syndrome (*MLH1*, *MSH2*, *MSH6*, *PMS2*), Familial Atypical Multiple Mole Melanoma (FAMMM; *CDKN2A*), Peutz–Jeghers syndrome (*STK11*), and Li-Fraumeni syndrome (*TP53*), each conferring variable PDAC risk [7] (Table 2). FPC refers to families with two or more first-degree relatives affected by PDAC in the absence of a known hereditary cancer syndrome. While a subset of FPC cases is attributable to pathogenic germline variants, the majority remain genetically unexplained, suggesting a polygenic or multifactorial etiology [13]. Hereditary pancreatitis, often caused by PRSS1 mutations, is associated with chronic inflammation of the pancreas beginning in childhood. The ongoing inflammatory milieu predisposes to malignant transformation over time. Although it represents a rare etiology, the cumulative lifetime risk of PDAC in hereditary pancreatitis can reach as high as 40%, underscoring the importance of early identification and tailored risk management [13,53]. To further aid in the clinical application of these concepts, Table 2 outlines the major genetic syndromes and conditions associated with increased risk of PDAC. It provides a concise overview of associated germline mutations, the spectrum of common cancers seen in each syndrome, established screening criteria, the recommended age to begin surveillance, and the estimated lifetime risk of PDAC.

Despite these elevated risks, disease penetrance is incomplete. Environmental modifiers, gene–environment interactions, and epigenetic factors may influence phenotypic expression, and not all mutation carriers will develop PDAC. Conversely, not all PDAC-affected families harbor identifiable germline mutations [54]. As such, pedigree analysis and genetic counseling are essential tools for identifying at-risk individuals and tailoring personalized surveillance.

**Table 2:** Genetic Syndromes and Conditions Associated with Increased Risk of Pancreatic Ductal Adenocarcinoma: Mutations, Screening Guidelines, and Risk Estimates. FDR: First Degree Relatives.

Syndrome	Germline Mutations	Common Cancers	Screening Criteria [13]	Recommended Age to Start Screening [13]	Lifetime Risk of PDAC [55]
**Hereditary Breast and Ovarian Cancer [56]**	BRCA1/2, PALB2	Breast, ovarian, prostate, and pancreatic	≥1 FDR or any two affected relatives	Age 50 or 10 years before the youngest affected	BRCA1: Relative Risk: 2.26; BRCA2: Relative Risk: 3.51
**Familial Atypical Multiple Mole/Melanoma [56,57]**	CDKN2A	Melanoma, pancreatic	None required (for CDKN2A, P16 variant)	Age 40 or 10 years earlier than the youngest affected relative	Cumulative Risk: 17% by age 75 years
**Peutz-Jeghers Syndrome [57]**	STK11	GI (colon, small bowel, stomach), breast, pancreatic, lung	None required	Age 35 or 10 years earlier than the youngest affected relative	Relative Risk: 132; Cumulative Risk: 2.4% at age 40, 3.9% at age 50, 11.1% at age 60, and 25.6% at age 70 years
**Li-Fraumeni Syndrome** [58]	TP53	Breast, brain, sarcoma, leukemia, adrenal, pancreatic	≥1 FDR or any two affected relatives	Age 50 or 10 years earlier than the youngest affected relative	Relative Risk: 7.73
**Lynch Syndrome** [58]	MLH1, MSH2, MSH6, PMS2	Colorectal, endometrial, ovarian, stomach, urinary tract, hepatobiliary, pancreatic	≥1 FDR or any two affected relatives	Age 50 or 10 years before the youngest affected	Relative Risk: 5–9; Cumulative Risk: 1.3% by age 50 years, 3.7% by age 70 years
**ATM Mutation**	ATM	Breast, pancreatic	≥1 FDR or any two affected relatives	Age 50 or 10 years earlier than the youngest affected relative	Relative Risk: 6.5
**Hereditary Pancreatitis**	PRSS1	Pancreatic	None required	Age 40	Standardized Incidence Ratio: 53 (95% CI: 23–105) Cumulative Risk: 40% by age 70 years
**Familial Pancreatic Cancer (FPC)**	Unknown	Pancreatic	≥2 FDR	Age 50 or 10 years earlier than the youngest affected relative	16–39% [53]

## 4. Role of Biomarkers in Early Detection

Recent advancements in technologies have facilitated the identification of biomarkers across various molecular levels, including genetic, transcriptomic, metabolomic, and proteomic domains. In high-risk individuals, biomarker based-tools may supplement current imaging surveillance protocols and improve patient stratification for earlier PDAC intervention [55].

The most established biomarker in routine PDAC care is CA19-9, which has demonstrated increased levels beginning up to two years before clinical diagnosis. Sensitivities of 60–68% with specificities of 90–95% have been reported within one year of PDAC diagnosis using CA19-9 alone [59]. Although CA19-9 has shown clinical utility for PDAC monitoring, it suffers from limited sensitivity and specificity in the early stages of disease. Several studies have shown that combining CA19-9 with other markers such as hemoglobin A1c (HbA1c), circulating tumor cells (CTCs), or exosomes substantially enhances its diagnostic performance [59,60]. One notable test under investigation (NCT03693378) is the IMMray PanCan-d assay (Immunovia), which integrates CA19-9 with eight serum biomarkers involved in hormone transport, complement, coagulation, and protease inhibition pathways. It is designed to stratify risk among high-risk individuals and identify those needing further diagnostic evaluation. Early data are promising, and the ongoing PanFAM-1 longitudinal study aims to validate its clinical application [61].

Other proteomic and metabolomic profiling studies have also yielded several promising candidate biomarkers. The PancRISK panel, which includes LYVE1, REG1B, and TFF1, demonstrated an area under the receiver operating curve (AUC) of 0.92 up to 1 year prior to diagnosis and 0.77 up to 2 years before PDAC detection [62,63]. A more recent 20-feature panel incorporating both proteomic and clinical features reached an AUC of 0.91 at 1 year with a sensitivity of 92% and an AUC of 0.85 at 2 years with a sensitivity of 61% prior to PDAC diagnosis [64]. Tumor-induced metabolic reprogramming in PDAC can be detected through plasma metabolites. Metabolic markers such as branched-chain amino acids (BCAAs), linked to tumor-driven catabolism and proteolysis, are elevated years prior to PDAC diagnosis, correlating with increased risk [65]. In addition, a case–control study by Mayerle et al. identified a metabolic profile that, when combined with CA19-9, achieved an AUC of 0.94 in distinguishing PDAC from CP, highlighting its potential role in addressing diagnostic ambiguity in high-risk individuals [60].

Circulating tumor DNA (ctDNA) and cell-free DNA (cfDNA), while showing a diagnostic accuracy of over 95% in detecting stage IV PDAC via analyzing methylation signatures of cfDNA [66], are often below detection thresholds in early-stage PDAC, limiting their current utility in routine screening [67]. However, the PATHFINDER study, utilizing a multi-cancer early detection test, identified over 90% stage I and stage IV PDAC cases [68]. Novel methods such as methylation analysis of cfDNA, including analyzing 5-hydroxymethylcytosine (5hmC) profiles, are being pursued to address this unmet need. For detecting early-stage (stage I/II) pancreatic cancer, it has shown a sensitivity of 68.3% (95% CI, 51.9–81.9%) and an overall specificity of 96.9% (95% CI, 96.1–97.7%) [69].

RNA biomarkers, including messenger RNA (mRNA), long noncoding RNAs (lncRNAs), microRNAs (miRNAs), and circular RNAs (circRNAs), have been increasingly investigated in body fluids. MiRNAs like miR-21, miR-10b, miR-221, miR-210, miR-155, and miR-196a are upregulated in early PDAC, while miR-34a/b, let-7, miR-146a, and miR-126 are downregulated, reflecting their involvement in key oncogenic pathways [70,71,72]. FKBP1A mRNA, detectable in circulating white blood cells, has also been proposed as an early detection marker [73]. LncRNAs such as HOTTIP, MALAT-1, MEG3, and CASC2 have been implicated in PDAC pathogenesis [74]. Furthermore, Li et al. identified a novel piRNA, piR-162725, which, when combined with the serum marker CA19-9, significantly improved the identification of PDAC patients [75].

Exosomes, small extracellular vesicles secreted by cells, have emerged as promising biomarkers for PDAC detection. Exosomes released from PDAC cells contain overexpressed RNAs such as WASF2, ARF6, SNORA74A, and SNORD25, with analysis showing AUC greater than 0.9 for early stages of PDAC [76]. Among these, levels of WASF2 showed the strongest correlation with the risk of pancreatic cancer [76]. Yu et al. developed a nano liquid biopsy assay capable of co-recognizing dual tumor exosome biomarkers, addressing previous limitations in specificity and sensitivity associated with traditional detection methods [77]. This innovative approach facilitates the detection of tumor-derived exosomes at ultralow concentrations, enhancing early diagnostic capabilities. Despite their promise, RNA biomarkers face challenges related to reproducibility, and large-scale validation remains essential.

Several non-traditional modalities are also being evaluated for PDAC biomarker discovery. Tumor-educated platelets (TEPs) have shown potential for harboring tumor-specific RNA signatures [78]. Volatile organic compounds (VOCs) detectable in exhaled breath have demonstrated sensitivity as high as 90% in symptomatic patients [79,80]. Clinical trials in the United Kingdom (NCT05727020) are currently underway to validate VOC biomarkers for cancer detection. Meanwhile, advances in paper-based nanodiagnostics, using gold nanoparticles or MiRNA-492 and MiRNA-495 on electrochemical sensors, are enabling highly sensitive platforms for detecting key tumor-derived molecules [81,82,83].

## 5. Imaging Modalities in PDAC Detection

Advances in cross-sectional imaging, particularly CT and MRI, positron emission tomography-CT (PET-CT), EUS, and molecular imaging, offer new avenues for non-invasive detection and staging of PDAC. In current clinical practice, contrast-enhanced multidetector CT (MDCT) using a dedicated pancreatic protocol with thin-slice, multiphasic imaging continues to serve as the first-line modality for diagnosis and staging [84,85]. MDCT provides high-resolution assessment for tumor extent, vascular involvement, and resectability, which are essential for guiding treatment decisions. CT also benefits from broad availability, lower cost, and a strong evidence base for surgical decision-making. While pancreas-protocol MDCT achieves a high sensitivity of approximately 89% for overt PDAC [86], its sensitivity declines sharply for small tumors (<2 cm), with estimates ranging from 70 to 80% [87]. In clinical scenarios where CT findings are equivocal or when patients have contraindications to iodinated contrast agents, contrast-enhanced MRI serves as a problem-solving tool [84,85]. MRI offers superior soft tissue contrast compared to CT, with the highest sensitivity and specificity (93% and 89%, respectively) amongst imaging modalities [88]. MRI is particularly advantageous for the detection of isoattenuating pancreatic tumors not well visualized on CT [85,89]. PET-CT is generally utilized as an adjunct to MDCT, particularly in patients at high risk for metastatic disease. Clinical scenarios where PET-CT can be considered include indeterminate findings on conventional imaging, bulky primary tumors, suspicious regional lymphadenopathy, markedly elevated CA19-9 levels, or disproportionate systemic symptoms. Additionally, PET-CT may be used to assess treatment response in patients undergoing systemic therapy [85].

EUS has become a preferred modality for surveillance in high-risk individuals and serves as a complementary tool alongside cross-sectional imaging. EUS provides excellent visualization and is particularly effective for detecting malignant strictures, periampullary masses, and subtle parenchymal changes. However, its diagnostic performance is highly operator-dependent, and standardization efforts have been proposed to enhance image acquisition and interpretation for PDAC detection [85,90]. In routine practice, EUS is mainly reserved for tissue acquisition via fine needle aspiration or biopsy due to its invasive nature, limited accessibility, and procedural complications, such as pancreatitis, perforation, and anesthesia-related risks [91].

Recent advances in EUS-based techniques—including contrast-enhanced EUS (CE-EUS), elastography, and needle-based confocal laser endomicroscopy (nCLE)—have demonstrated potential in improving early detection of PDAC, though further validation is needed before widespread clinical implementation. CE-EUS has shown higher sensitivity than conventional imaging for small lesions (<20 mm); in a retrospective single-center study, CE-EUS achieved 95% sensitivity for tumors 11–20 mm and 70% for tumors ≤ 10 mm, outperforming CT and MRI in both categories [92]. However, a multicenter randomized trial found no significant difference in diagnostic sensitivity between CE-EUS-guided and conventional EUS-guided biopsy for solid pancreatic lesions, suggesting that CE-EUS may be selectively useful for small, indeterminate lesions rather than for routine use [93]. EUS elastography has also emerged as a potentially sensitive modality for characterizing small solid pancreatic lesions. A single-center retrospective study reported a 100% negative predictive value for ruling out malignancy in lesions ≤ 20 mm without main pancreatic duct dilation [94]. In another prospective single-center study, combined qualitative and quantitative elastographic parameters yielded 99% sensitivity and 94.6% specificity [95]. Needle-based confocal laser endomicroscopy (nCLE) has shown improved diagnostic accuracy for pancreatic cystic lesions. A prospective single-center study reported 98% sensitivity and 94% specificity for identifying mucinous cysts, outperforming standard cyst fluid analysis [96]. A multicenter study further demonstrated 80% sensitivity and 100% specificity in distinguishing mucinous from non-mucinous cysts [97]. Collectively, these advanced EUS modalities may offer improved diagnostic precision for early pancreatic cancer and its precursors, but remain investigational and require further multicenter validation before routine clinical adoption.

Advanced MRI techniques, including diffusion-weighted imaging (DWI), intravoxel incoherent motion (IVIM) imaging, dynamic contrast-enhanced MRI (DCE-MRI), hyperpolarized MRI, and magnetic resonance elastography (MRE), offer potential in enhancing the detection and characterization of PDAC. DWI assesses tissue cellularity and has demonstrated high diagnostic performance in detecting PDAC, with sensitivity and specificity exceeding 95% [98]. DWI also plays a crucial role in detecting liver metastases and predicting lesion aggressiveness [99]. IVIM analysis allows separation of diffusion and perfusion components, offering improved characterization of malignant vs. benign pancreatic lesions without the need for contrast agents [100]. DCE-MRI evaluates tissue perfusion and hypoxia, providing insights into tumor aggressiveness. Hyperpolarized pyruvate MRI detects metabolic changes, particularly the alanine-to-lactate ratio in precursor lesions and PDAC [101,102,103,104], which may aid in early diagnosis and monitoring treatment response. MRE and tomoelastography provide mechanical characterization of the tissue and quantify tissue stiffness and fluidity, offering a non-invasive assessment of tumor fibrosis. These modalities have demonstrated potential in distinguishing PDAC from autoimmune pancreatitis, with PDAC exhibiting increased tissue stiffness not only within the tumor but also in the surrounding pancreatic parenchyma [105]. Taken together, these advanced MRI techniques hold promise as adjuncts to conventional imaging in PDAC evaluation. However, further multicenter studies and standardization are necessary before their routine clinical implementation.

## 6. Artificial Intelligence Aided Tools in PDAC Detection

AI offers a promising avenue for the early detection of PDAC, particularly through deep learning and radiomics approaches applied to imaging and clinical data [106]. A recent scoping review of 30 studies found that AI was most often used for diagnosis and risk prediction (47%), with convolutional neural networks being the most frequently used algorithm (60%) [106]. These models commonly relied on CT, MRI, and EUS images, achieved high diagnostic accuracy, with reported pooled sensitivity and specificity of 89.5% and 89.9%, respectively [106].

Radiomics has emerged as an innovative methodology. Radiomics enables the extraction of high-dimensional quantitative features that are not discernible to the human eye, such as Gray-Level Co-Occurrence Matrix (GLCM), Gray-Level Dependence Matrix (GLDM), and Gray-Level Size Zone Matrix (GLSZM), which have been used to detect early-stage PDAC [107,108,109,110]. These are texture analysis features that quantify spatial relationships and intensity patterns within imaging data—for example, GLCM measures how often pairs of pixel intensities occur in a specified spatial relationship, while GLDM and GLSZM capture dependencies and size zones of homogeneous intensity regions. Such features provide a quantitative fingerprint of tissue heterogeneity, allowing radiomics-based models to detect subtle tumor characteristics that may escape human observation. Radiomics-based machine learning (ML) models have demonstrated the ability to distinguish PDAC from normal pancreas with diagnostic accuracies ranging from 86.5% to 99.2% [111]. By capturing subtle morphologic and textural changes, radiomics-based analysis enables earlier and more accurate risk stratification of PDAC [112]. For example, Chen et al. and Chu et al. developed radiomics algorithms that achieved high sensitivity without requiring detailed manual segmentation of tumors [113,114]. Gotta et al. further proposed that fusing radiomics with dual-energy CT (DECT) could highlight subtle tumor heterogeneity, achieving diagnostic accuracies of 98% in arterial phase imaging [115]. Wang et al. identified 91 stable radiomics features capable of differentiating precursor stage malignancy from various chronic pancreatic conditions [116]. Moreover, Wang et al. highlighted the utility of multitasking dynamic contrast-enhanced MRI (DCE-MRI) in differentiating PDAC from CP. Their quantitative approach analyzing microcirculation parameters (e.g., blood flow, plasma volume fraction, transfer constant, and extracellular volume fraction) achieved high diagnostic accuracy (AUC 0.821), distinguishing CP from PDAC, surpassing conventional imaging techniques [117].

In addition to diagnostic applications, recent efforts have also focused on risk prediction using prediagnostic imaging. For instance, Qureshi et al. demonstrated that radiomic analysis of abdominal CT scans obtained 6 months to 3 years prior to PDAC diagnosis could unveil micro-level morphological and textural changes not apparent to human readers. These features enabled a naïve Bayes classifier to stratify individuals at high risk of developing PDAC, achieving an external validation accuracy of 86% [118]. Building on this work, Javed et al. developed a subregional AI model that further improved performance by identifying localized precancerous changes within specific pancreatic regions. Their model attained an accuracy of 89.3%, with sensitivity of 86% and specificity of 93%, notably surpassing the performance of models based on whole-pancreas analysis [119]. These studies highlight the potential of combining abdominal CT imaging with AI techniques to proactively identify individuals at increased risk for PDAC, thereby enabling earlier surveillance before tumor formation [112]. Beyond imaging, ML models such as logistic regression, vector machines, decision trees, and k-means clustering have shown high classification accuracies, exceeding 99.5% in some studies [120].

In addition to image-based models, several efforts are focused on mining electronic health records (EHRs) to identify individuals at increased risk for PDAC using structured and longitudinal clinical data. For instance, a recent study developed machine learning-based clinical prediction models across two healthcare systems, relying on well-established parameters such as age, glycated hemoglobin (HbA1c), ALT, weight change, and abdominal pain. This approach extended risk stratification to a broader, population-level setting beyond high-risk groups with familial or genetic predisposition. The study demonstrated the feasibility of leveraging EHR data to target surveillance strategies for sporadic PDAC [121]. Another study by Placido et al. applied deep learning to over 9 million patient records from Denmark and the US Veterans Affairs system. By analyzing temporal patterns of diagnosis codes, their model—CancerRiskNet—achieved an area under the receiver operating characteristic curve (AUROC) of up to 0.88 for predicting PDAC within 36 months, demonstrating strong potential for early identification based on longitudinal clinical trajectories [122]. Meanwhile, biomarkers—including ctDNA, exosomal RNA, and proteomic signatures—are combined with AI platforms to enhance detection accuracy. For example, CancerSEEK is a blood test designed to detect multiple cancer types through assessment of circulating proteins and mutations in cell-free DNA. CancerSEEK achieved a sensitivity of around 70% for pancreatic cancer [123]. In addition, the gut and oral microbiome have emerged as novel biosources for AI-based early detection tools. Microbial signatures in fecal or salivary samples—analyzed via machine learning—can distinguish PDAC from controls with high specificity. More recently, shotgun metagenomic and 16S rRNA sequencing revealed that fecal-based classifiers achieved an area under the receiver operating characteristic curve (AUROC) of up to 0.95 when combined with serum CA19-9 levels [124]. The AUROC summarizes a model’s ability to distinguish between classes across all threshold values, with values closer to 1.0 indicating excellent discriminative performance. In addition to high sensitivity, the classifier’s specificity was also tested against a metagenomic study in a healthy population [124].

Despite these advances, several limitations remain, including small dataset sizes, lack of external validation, variability in preprocessing methods, and inconsistent adherence to AI reproducibility standards. Only two studies utilized AI guidelines, such as CLAIM or TRIPOD-AI, which help standardize study quality and ensure methodological rigor. There was also insufficient sharing of code, data, and training models. These limitations can decrease reproducibility [111]. The importance of open, transparent, and standardized practices should be emphasized in AI-aided tools. Data privacy is also a major concern, as radiomics depends on large imaging datasets to train the model. These images may contain sensitive patient information, requiring strict anonymization and secure data-sharing protocols [111]. Nowadays, many AI tools have demonstrated accuracy in distinguishing PDAC vs. non-PDAC, but they have not demonstrated clinical significance by predicting clinical outcomes and prognosis [112]. Nonetheless, AI holds substantial potential to enhance early PDAC diagnosis, especially when integrated with high-resolution imaging and structured clinical data in multicenter, standardized research settings [111].

## 7. Conclusions and Future Directions

Early detection of PDAC remains a major clinical challenge, but meaningful progress has been made through the identification of high-risk individuals, the development of biomarker platforms, and advances in imaging technologies. The integration of artificial intelligence, liquid biopsy, microbiome profiling, and high-resolution imaging has paved the way for a new era of precision diagnostics, enabling the possibility of detecting PDAC at a curable stage.

Moving forward, the most promising strategies will likely depend on the integration of multimodal data sources, including genetic, metabolic, imaging, and molecular biomarkers, into refined risk prediction models that can help identify at-risk individuals and promote longitudinal surveillance. These models must be complemented by highly specific and sensitive diagnostic assays and followed by confirmatory imaging to detect subclinical tumors. Despite advances in recent years, the challenges persist. Translating emerging tools into clinical practice will require continued investment in prospective clinical studies, rigorous validation of predictive tools across diverse populations, and interdisciplinary collaboration.

Key priorities for advancing the field include:

- Develop integrated, data-driven risk stratification frameworks: Combine clinical, metabolic, and genetic factors with AI-enhanced EHR models to identify individuals at highest risk, such as those with new-onset diabetes, familial predisposition, or pancreatic lesions. Initiate prospective trials of prevention and monitoring in these groups to evaluate the impact of early detection strategies.
- Validate and implement multimodal early detection strategies: Coordinate efforts to evaluate composite biomarker panels (e.g., ctDNA, exosomes, proteomics, methylation) alongside imaging-based tools such as radiomics and machine learning–assisted interpretation, within longitudinal surveillance cohorts.
- Elucidate biological mechanisms of early tumorigenesis and tissue crosstalk: Investigate how metabolic dysfunction, pancreatic stellate cell signaling, inflammation, and endocrine-exocrine interactions contribute to PDAC initiation. Use preclinical models to test low-toxicity agents that may interrupt or reverse early tumorigenesis, enabling rational chemoprevention strategies.
- Design adaptive, personalized surveillance protocols: Shift from static screening intervals to dynamic, risk-responsive surveillance approaches informed by changes in biomarkers, imaging findings, and clinical variables.
- Strengthen collaborative infrastructure to enable translational research: Build multi-institutional cohorts, leverage organoid and co-culture systems, and support federated data-sharing platforms and interdisciplinary networks to accelerate the clinical implementation of early detection tools.

With sustained and coordinated effort in these areas, the field is poised to transform PDAC from a disease of late-stage detection to one that is actionable and manageable at its earliest and most treatable stages.
